# Supplementary material for: Violence against older women: a protocol for a systematic review of qualitative literature
Source: BMJ Open. 2019 May 28;9(5):e028809. doi: 10.1136/bmjopen-2018-028809 (PMC6550033; doi:10.1136/bmjopen-2018-028809)
Supplement: Supplementary data [file bmjopen-2018-028809supp001.pdf]

PubMed search

1. **Concept #1: Age – 50 and above**

Aged [MeSH Terms] OR Frail Elderly [MeSH Terms] OR senior* [TIAB] OR elderly [TIAB] OR Older [TIAB] OR later life [TIAB] OR above 50 [TIAB] OR above 60 [TIAB]

2. **Concept #2: Women**

Women [MeSH Terms] OR female [MeSH Terms] OR wife [TIAB] OR spouses [MeSH] OR wives [TIAB] OR "female partners" [TIAB] OR spouse [TIAB] OR spouses [TIAB]

3. **Concept #3: Violence**

Elder abuse [MeSH Terms] OR domestic violence [MeSH Terms] OR Intimate Partner Violence [MeSH Terms] OR battered women [MeSH] violence [MeSH Terms] OR aggression [MeSH Terms] OR spouse abuse [MeSH Terms] OR Physical Abuse [MeSH Terms] OR Rape [MeSH] OR elder neglect [TIAB] OR elder mistreatment [TIAB] OR elder maltreatment [TIAB] OR assault [TIAB] OR sexual abuse [TIAB] OR sexual assault [TIAB] psychological abuse [TIAB] or psychological violence [TIAB] OR emotional abuse [TIAB] or emotional violence [TIAB] or neglect [TIAB] or economic abuse [TIAB] or verbal abuse [TIAB] or violence against women [TIAB]

4. **Concept #4: Methodology**

(qualitative research methodology filter adapted from http://libguides.sph.uth.tmc.edu/search_filters/pubmed_filters and http://guides.lib.uw.edu/hsl/qualres/pubmed)

Qualitative research [MeSH Terms] OR Interviews as Topic [MeSH Terms] OR Focus Groups [MeSH Terms] OR Grounded Theory [MeSH Terms] OR Nursing Methodology Research [MeSH Terms] OR Anecdotes as Topic [MeSH Terms] OR Narration [MeSH Terms] OR Video Recording [MeSH Terms] OR Tape Recording [MeSH Terms] OR Personal Narratives as Topic [MeSH Terms] OR "semi-structured" [TIAB] OR semistructured [TIAB] OR unstructured [TIAB] OR structured [TIAB] OR informal [TIAB] OR "in-depth" [TIAB] OR indepth [TIAB] OR "face-to-face" [TIAB] OR "focus group" [TIAB] OR "focus groups" [TIAB] OR qualitative [TIAB] OR ethnograph* [TIAB] OR "key informant"[TIAB] OR thematic [TIAB] OR phenomenol* [TIAB] OR "grounded theory" [TIAB] OR "grounded study" [TIAB] OR "grounded studies" [TIAB] OR "grounded research" [TIAB] OR "grounded analysis"[TIAB] OR "grounded analyses"[TIAB] OR "life story" [TIAB] OR "life stories" [TIAB] OR "participant observation"[TIAB] OR "action research"[TIAB] OR "purposive sampling"[TIAB] OR "content analysis" [TIAB] OR discourse [TIAB] OR "narrative analysis" [TIAB]

5. **Combine: #1 AND #2 AND #4**

6. **Combine: #2 AND #3 AND #4**
