# Supplementary material for: Violence against older women: a protocol for a systematic review of qualitative literature
Source: BMJ Open. 2019 May 28;9(5):e028809. doi: 10.1136/bmjopen-2018-028809 (PMC6550033; doi:10.1136/bmjopen-2018-028809)
Supplement: Author's manuscript [file bmjopen-2018-028809.draft_revisions.pdf]

BMJ Open is committed to open peer review. As part of this commitment we make the peer review history of every article we publish publicly available.

When an article is published we post the peer reviewers' comments and the authors' responses online. We also post the versions of the paper that were used during peer review. These are the versions that the peer review comments apply to.

The versions of the paper that follow are the versions that were submitted during the peer review process. They are not the versions of record or the final published versions. They should not be cited or distributed as the published version of this manuscript.

BMJ Open is an open access journal and the full, final, typeset and author-corrected version of record of the manuscript is available on our site with no access controls, subscription charges or pay-per-view fees (http://bmjopen.bmj.com).

If you have any questions on BMJ Open's open peer review process please email info.bmjopen@bmj.com

BMJ Open

Violence against older women: A protocol for a systematic review of qualitative literature

Journal:	*BMJ Open*
Manuscript ID	bmjopen-2018-028809
Article Type:	Protocol
Date Submitted by the Author:	29-Dec-2018
Complete List of Authors:	Meyer, Sarah; World Health Organization, Department of Reproductive Health and Research<br>Lasater, Molly; Johns Hopkins University Bloomberg School of Public Health, Mental Health<br>Garcia-Moreno, Claudia; World Health Organisation, Department of Reproductive Health and Research
Keywords:	violence, older women, systematic review

SCHOLARONE™
Manuscripts

Violence against older women: A protocol for a systematic review of qualitative literature

Sarah R. Meyer[1], Molly Lasater[2], Claudia García-Moreno[1]

1. Department of Reproductive Health and Research, World Health Organization, Geneva, Switzerland.
2. Department of Mental Health, Johns Hopkins School of Public Health, Baltimore, USA.

Corresponding author: Dr Sarah Meyer, Department of Reproductive Health and Research, World Health Organization, smeyer@who.int, Phone number: 0041 22 791 2111, Fax number: 0041 22 791 4171

Key words: violence against women, systematic review, qualitative research,

Word count: 3,756

For peer review only

Abstract:

Introduction: There is sparse evidence globally concerning patterns of and types of violence against women aged 50 and older, and this gap needs to be filled. Improved understanding of older women's experiences of violence, including types of violence, perpetrators, and health impacts, is needed to address evident gaps in the literature, address requirements for monitoring and reporting on global Sustainable Development Goal indicators, and inform policy and programming for preventing and responding to violence against older women. The aim of the systematic review is to identify, evaluate and synthesize qualitative studies from all countries, exploring violence against women aged 50 and above, identifying types and patterns of violence, perpetrators of violence, and impacts of violence on various health outcomes for older women.

Methods and analysis:

A systematic search for qualitative studies of violence against older women will be conducted in the following databases: PubMed, PsycINFO, Embase, CINAHL, PILOTS, ERIC, Social Work Abstracts, International Bibliography of the Social Sciences, Social Services Abstracts, ProQuest Criminal Justice, and Dissertations & Theses Global. Studies will be focused on violence against older women (aged 50 and above), using qualitative methodology, exploring women's experiences of any type of violence perpetrated by any type of perpetrator. Two authors will independently review titles and abstracts retrieved through the search strategy, to determine which should be included for full text review. Data extraction will be conducted independently by one author and quality assessment will be conducted by two authors, using an adapted version of the Critical Appraisal Skills Programme [CASP] scale. Data will be analysed and synthesised using a thematic synthesis approach.

Dissemination:

Findings will be disseminated through a publication in a peer-reviewed journal and used to inform development of a module to measure violence against older women, for use in specialized violence against women surveys.

Strengths and limitations of this study:

- This systematic review is designed with a comprehensive search strategy, to allow inclusion of all relevant qualitative studies of violence against older women globally;
- This systematic review builds on strengths and addresses limitations of previous qualitative and quantitative systematic reviews of violence against older women; and

For peer review only

- This review focuses on qualitative literature, in order to build understanding of lived experiences of older women subjected to different forms of violence.

Introduction:

Violence against women [VAW] is a major public health problem, a gender inequality issue and a human rights violation. Violence has significant and long-lasting impacts on women's physical and mental health, including injuries, unintended pregnancy, adverse birth outcomes, abortions (often in unsafe conditions), HIV and sexually transmitted infections, depression, alcohol-use disorders and other mental health problems[1-5].

Much of this research has focused on women of reproductive age (15-49 years) as they suffer the brunt of partner violence and sexual violence. However, there is sparse evidence globally concerning patterns of and types of violence against women aged 50 and older[6], and this gap needs to be filled. A global research priority setting exercise on interpersonal violence indicated widespread consensus regarding the limited understanding of violence against older women. The exercise identified description of the nature, magnitude, distribution and consequences of elder abuse as priority research areas[7]. Specific risk and protective factors for violence victimization amongst women of reproductive age may not be relevant or applicable in the case of older women. For older women, different relationship dynamics may influence forms of abuse[8 9], recent exposure to violence may be interlinked with violence victimization at different stages of the lifecourse[10 11], and dynamics of ageing may influence decisions to disclose or report abuse[12]. Current lack of data on violence against older women may negatively impact service development and provision, including gaps in services, limited understanding of barriers to reporting and help-seeking amongst older women who are subjected to violence. It may also lead to inappropriate policy or programmatic responses to violence[10 13]. International policy frameworks, including the Madrid International Plan of Action on Ageing specifically identifies elimination of violence, neglect and abuse of older women as a priority, recognizing that older women's vulnerabilities are compounded by societal discrimination, poverty and lack of access to legal protections[14].

There are currently two dominant theoretical frameworks to understanding violence against older women, each linked to different definitions of violence and assumptions regarding measurement, prevention of violence and other interventions: older adult mistreatment and older adult protection[15 16 6]. The older adult mistreatment framework

For peer review only

is informed by social gereontology, and understands violence as forms of elder abuse – focusing on age as the primary vulnerability to exposure to violence. The older adult protection framework specifically understands violence within the context of care-giving and institutional arrangements, where older adults' vulnerability to violence is a result of reliance on caregivers. There is also an approach that focuses specifically on one form of violence affecting older women, namely intimate partner violence [IPV]. This approach adopts the definition of IPV used for women and girls below 50, and seeks to expand understanding of sexual, physical and psychological violence perpetrated by partners that older women may experience.

The lack of an over-arching framework for understanding violence against older women has resulted in literature and evidence that is fragmented, with some research focusing only on specific types of violence against older women (e.g., IPV)[17], some research (in particular, using the older adult mistreatment framework) lacking a focus on the gendered dimensions of violence[16 18], and some approaches that focus specifically on older women in protective settings and relationships with caregivers rather than also including women in community settings. A 2013 United Nations report on neglect, abuse and violence against older women noted that these divergent theoretical frameworks have stymied data collection and evidence generation efforts; for example, some studies (in particular, population-based studies of IPV) have focused on women of reproductive age, while other studies (for example, studies of women in care-settings) have excluded measurement of some types of violence, for example, perpetrated by intimate partners[15]. The elder abuse perspective has traditionally lacked understanding of the gendered nature, i.e., differences between women and men, of age-related vulnerabilities, for example, that women are less likely to have adequate pensions than men and that older women are more likely than older men to be financially dependent on family members[6 19 20]. Some researchers note that the term "elder abuse" may homogenise "older people rather than recognising individual differences, including gender"[21]. In the study of IPV, research has primarily focused on women of reproductive age, potentially leading to marginalization of older women's experiences of IPV, which may differ in type and nature[12]. Researchers note that this conceptual split has resulted in "lack of attention of ageing issues in research on violence against women and, conversely, the lack of gender-based analysis in elder abuse research"[22]. Further, there is concern that these approaches primarily respond to the situation of older women in high-income, industrialized settings, and that forms of violence and vulnerability to violence more prevalent in low-income contexts are not adequately reflected in current literature, theoretical approaches and measurement methods[15 22]. Research is spread

For peer review only

between disciplines and fields of inquiry, yet a more comprehensive understanding of violence against older women can be built by bridging these different approaches,[23] incorporating a perspective that is both age and gender-responsive[12]. Improved understanding of older women's experiences of violence, including types of violence, perpetrators, and health impacts, is needed to address evident gaps in the literature, address requirements for monitoring and reporting on global SDG indicators, and inform policy and programming for preventing and responding to violence against older women.

This manuscript describes a protocol for a systematic review that complements a previous systematic review of quantitative studies of elder abuse (against men and women aged 60+). That review found the global prevalence of elder abuse in community settings of men and women is 15.7% in the past year, with psychological abuse and financial abuse the most prevalent forms of abuse reported. The studies were very heterogenous and methodological variables were associated with prevalence, with larger sample size and random sampling associated with lower prevalence rates[24]. Sex was not found to be significantly associated with prevalence rates in the review, however, some studies did not provide sex-specific analysis. The prevalence of elder abuse in institutional settings may be as high as 64.2%, with data obtained from self-reports of perpetration by care-givers in institutions, with prevalence estimates highest for psychological abuse, and physical violence, neglect, financial abuse and sexual abuse less prevalent[25]. Findings from institutional settings indicate that being female is a significant risk factor for vulnerability to abuse. Analysis from the same review which focused on the findings specific to abuse of older women found a global prevalence of 14.1%, and similar to the analysis of men and women, psychological abuse was the most prevalent form of violence, followed by neglect. Financial abuse was less prevalent amongst women than in the analysis including both men and women[22]. Overall, there has been more work identifying and synthesizing the quantitative literature on elder abuse and violence against women[22 26], which may miss out several forms of abuse older women are exposed to, particularly in low- and middle-income countries. This confirms a need to rigorously synthesize the qualitative literature.

There are some existing systematic reviews relevant to this one; those reviews primarily focus on specific forms of violence against older persons or women, and some focus on qualitative literature. Warmling et al. conducted a systematic review of IPV against "elderly men and women", focusing on prevalence studies (cross-sectional, population-based studies) in any country and exploring factors associated with the experience of

For peer review only

violence by men and women [18]. The exact age range constituting "elderly men and women" was not defined in the review. Findings included that psychological violence and economic abuse were the most prevalent forms of partner violence against older men and women, and alcohol-use, depression, low income, functional impairment and previous exposure to violence were associated with this violence amongst older men and women. An empirical review of IPV in later life used a qualitative coding scheme to identify theoretical frameworks, conceptual themes and methodological approaches in the existing literature, examining 27 quantitative, 22 qualitative and 7 mixed-methods studies[17]. The review identified controlling behaviours and power dynamics in relationships that continue between intimate partners into later life, and may become further entrenched by caregiving dynamics. Similarly to the previous reviews, findings included that forms of IPV in later life shifted from a higher prevalence of physical and sexual abuse during reproductive age, to a higher prevalence of forms of psychological abuse, with results indicating "a shift from physical to non-physical forms of violence dominated late-life scenarios."[22] A review of qualitative research on IPV amongst older women identified a number of themes relevant to the dynamics of IPV against older women [27]. For example, women described patterns of abuse that were continuous and consistent with previous experiences of abuse in families of origin and previous relationships. However, patterns and types of IPV against older women were also described as shifting from predominantly physical violence experienced previously, to neglect, psychological violence and economic abuse becoming more prevalent. A systematic review and meta-synthesis of qualitative studies of IPV and older women focused on how previous exposure to IPV influenced health-seeking behaviours, specifically in the area of mental health care[28]. Bows conducted an empirical review of sexual violence against older people, including qualitative and quantitative studies focused specifically on sexual violence, with studies including any population of older adults (age range not defined), identifying widespread variation in prevalence rates across studies, with higher levels of sexual violence identified in studies using a domestic violence framework. The role of socio-demographic factors, such as ethnicity, marital status and living arrangements, were not consistently associated with violence victimization, and studies identified a range of perpetrators, primarily intimate partners or adult children[23]. Amongst these existing systematic reviews of qualitative literature, none have focused specifically on older women, while being inclusive of any form of violence.

The present systematic review will build on previous systematic reviews and strengthen the evidence-base by i) focusing specifically on women, or if including studies with

For peer review only

women and men, only including studies that provide sex-stratified analyses; ii) focusing on *any* form of violence against women, rather than adopting a specific theoretical framework on what types of violence or perpetrators should be included from the outset iii) focusing on women aged 50 and above as many surveys often specifically focus on women up to 49 years of age; and iv) focusing specifically on qualitative studies, to explore the nature and dynamics of violence against older women from the perspective of women and in low- and middle-income countries.

Aim:

The aim of this systematic review is to identify, evaluate and synthesize qualitative studies from all countries, exploring violence against women aged 50 and above, identifying types and patterns of violence, perpetrators of violence, and impacts of violence on various health outcomes for older women.

Methods:

Search strategy

We identified the following domains as part of the research question: age (50 and above); women; violence; and qualitative approach. For each of these domains, we identified the relevant keywords and search terms, which vary by database. Search strategy has been appropriately modified for each database, including syntax and specific terms, topics and/ or headings.

We will not limit the search by year of publication, language of publication or type of publication at the stage of searching the databases. We have consulted with a librarian to provide input into the tailored search strategy that we have developed for each database, and the search strategy has been informed by other relevant systematic reviews and established approaches to identifying qualitative literature. The finalized search strategy for PubMed is included in Supplementary Appendix 1.

We will also search reference sections of relevant existing systematic reviews to identify articles that fit inclusion and exclusion criteria. We have also identified experts in the field, including researchers, practitioners and policy makers, and have contacted them to provide any relevant literature. All experts will be contacted at least twice to provide the research team with additional resources to consider for inclusion.

For peer review only

Data sources

We will conduct searches in the following electronic databases: PubMed, PsycINFO, Embase, CINAHL, PILOTS, ERIC, Social Work Abstracts, International Bibliography of the Social Sciences, Social Services Abstracts, ProQuest Criminal Justice, and Dissertations & Theses Global.

Data collection and analysis:

Eligibility criteria for the studies:

The inclusion criteria for studies to be considered to be included in this review are:

Inclusion criteria:

i. Women aged 50 and older (studies including men or including women younger than 50 will be included if sex-specific and age-specific analyses respectively are included. This age range was selected because 15-49 year is generally considered reproductive age and most current data and evidence on intimate partner violence is in this age group;
ii. Qualitative methodology (mixed methods studies will be included if the qualitative data analysis is presented separately); and
iii. Studies focused on women's experiences of **any type of violence** perpetrated by **any type of perpetrator.**

There are no language or date limits. The abstracts of articles not in English will be reviewed by team members fluent in that language where feasible.

Exclusion criteria:

i. Whole sample is children or adolescents (under 50 years)
ii. Sample of only men
iii. Only quantitative methodology
iv. Only includes perspective on violence against women reported by care providers, health professionals, nursing home managers.

Data management:

We will use EndNote V.X7.8 as our bibliographic software management platform. We will remove duplicates using EndNote, prior to exporting titles and abstracts to an Excel spreadsheet for review. Data extraction and quality assessment results will be recorded in separate Excel spreadsheets. A flow diagram will be presented in any final publications, showing results of each stage of the review and adhering to the PRISMA statement.

For peer review only

Selection of studies:

Two authors (SM, ML) will independently review titles and abstracts retrieved through the search strategy, to determine which should be included for full text review. If an abstract or title is considered relevant by either of the authors, it will be included for full text review. Two authors will independently review all articles selected for full text review for eligibility, to reach consensus on inclusion in the review. Any discrepancies will be resolved with the input of the third author (CGM). Reasons for excluding articles will be recorded.

Data extraction

After full text review, the following data will be extracted from all included articles using a standardized data extraction form: country studied, study design, research questions, sample size, characteristics of sample, data collection and analysis methods, main findings (as reported by the study's own authors), and any reported study limitations. Categories specific to the topic will also be included as data extraction progresses, including types and nature of violence, perpetrators of violence, and reported impacts of violence.

Data extraction will be conducted independently by one author, and accuracy of the data extraction checked by a second author, with discrepancies resolved by discussion to reach consensus. Reviewers will develop and pre-test a data extraction spreadsheet, to be used to compile a summary of characteristics and key findings of the included studies. The spreadsheet will also include categories relevant to data synthesis, described further below.

Quality appraisal

Risk of bias (quality) assessment:

Included studies will be assessed for quality using an adapted version of the Critical Appraisal Skills Programme [CASP] scale. The adapted scale, relevant for review of qualitative studies, will include the following domains: research aims, methodology, research design, recruitment strategy, data collection, data analysis, reflexivity, ethical considerations, findings, and research contributions [29]. The tool includes the following questions [30]:

1. Are the setting(s) and context described adequately?
2. Is the sampling strategy described, and is this appropriate?
3. Is the data collection strategy described and justified?

For peer review only

4. Is the data analysis described, and is this appropriate?
5. Are the claims made/findings supported by sufficient evidence?
6. Is there evidence of reflexivity?
7. Does the study demonstrate sensitivity to ethical concerns?
8. Any other concerns?

Two authors (SM, ML) will assess the quality of the studies and disagreement will be resolved either by discussion or inputs from the third reviewer (CGM). Studies will be assessed as having an overall quality of “high,” “medium” or “low” based on evaluation according to the CASP categories. Quality assessment will not be used to determine if any studies should be excluded, but rather to assess the strength of each study and as part of the confidence assessment (see below).

Strategy for data synthesis

Findings will be presented and synthesized using a thematic synthesis approach. Following some descriptive analysis (for example, number of studies examining specific types of violence, which types of risks and outcomes are described in studies), two authors will utilize the findings extracted as part of the data extraction process to conduct initial open coding and develop broad themes to use to further sort the findings. The authors will use axial coding of the text units in order to develop first, second and third order themes [31 32]. The synthesis and all aspects of the systematic review process will be reported following the 21-item checklist provided in the Enhancing Transparency in Reporting the Synthesis of Qualitative Research statement[33].

Patient and public involvement:

Patients were not involved in the development of this systematic review. Public were not consulted specifically for the development of the research questions, however, previous research and consultations with experts has indicated that this is a fruitful and important area of research in the field of violence against women research.

Discussion:

This manuscript provides a description of a protocol for a systematic review of qualitative literature on violence against older women (aged 50 and above). Strengths of the proposed review include utilization of multiple databases and search strategies to ensure inclusion of as much of the relevant literature as possible; a clear and structured process of data extraction and quality assessment; and transparent method of data synthesis, adhering to guidelines on systematic reviews of qualitative literature[33]. This

For peer review only

systematic review of qualitative literature focuses on violence against older women, including any form of violence, and including studies conducted globally. It is an important complement to existing systematic reviews on the same and related topics, which have primarily included quantitative literature or focused only on IPV. Limitations include that studies that do not include perspectives of women affected by violence themselves are excluded. There is a significant literature documenting the perspectives of care-givers and health-care professionals on elder abuse and other forms of violence against women, however, to ensure a focus on the lived experiences of women subjected to violence, we will exclude studies that only include perspectives of caregivers and health care professionals.

There are two Sustainable Development Goal [SDG] Indicators directly related to violence against women: 5.2.1, which measures intimate partner violence [IPV] (Proportion of ever-partnered women and girls aged 15 years and older subjected to physical, sexual or psychological violence by a current or former intimate partner in the previous 12 months), and 5.2.2, which measures non-partner sexual violence [NPSV] (Proportion of women and girls aged 15 years and older subjected to sexual violence by persons other than an intimate partner in the previous 12 months). VAW prevalence studies, whether implemented as stand-alone or using a module as part of a Demographic and Health Survey, Reproductive Health or other type of survey, have primarily included women of reproductive age (15-49). Yet valid monitoring of and reporting on these SDG indicators requires data on older women to be collected and reported systematically. Some recent data collection efforts have sought to address this gap in data and evidence by including women aged 50 and above in studies using these standard approaches to measuring violence against women (for example, the national prevalence studies in Bhutan and Laos[34 35]). However, this approach to measurement of violence against older women – utilizing standard VAW survey measures, – may not adequately capture the specific violence experiences of older women. Women aged 50 and above may experience different types and patterns of violence, with risk factors and impacts on physical and mental health, that differ compared to women aged below 50. This systematic review will inform efforts to increase and improve availability and quality of data on violence against women aged 50 and above. This includes, for example, efforts led by the World Health Organization to develop or adapt a quantitative module to measure violence against older women. When we complete the systematic review, we will develop and refine a set of domains and themes that emerged from the data, to inform development of the module.

For peer review only

Dissemination:

We will present findings in a manuscript for publication in a peer-reviewed journal, through coordinated dissemination to researchers, practitioners, data users and generators with an interest in violence against women, and to experts selected to participate in an Expert Meeting on Violence against Older Women, to be convened by the Department of Reproductive Health and Research, World Health Organization.

Contribution: SM and CGM conceptualized and developed the systematic review. SM drafted the manuscript. ML and CGM read and provided substantive feedback on all sections of the manuscript.

Competing interest: There are no competing interests.

Funding: The systematic review is funded by the Department for International Development, UNWomen-World Health Organization Joint Programme on Strengthening Methodologies and Measurement and building national capacities for Violence against Women data

References:

1. WHO. Global and regional estimates of violence against women: prevalence and health effects of intimate partner violence and non-partner sexual violence. Geneva: WHO 2013.
2. Ellsberg M, Jansen HA, Heise L, et al. Intimate partner violence and women's physical and mental health in the WHO multi-country study on women's health and domestic violence: an observational study. *Lancet (London, England)* 2008;371(9619):1165-72. doi: 10.1016/s0140-6736(08)60522-x [published Online First: 2008/04/09]
3. Valladares E, Ellsberg M, Pena R, et al. Physical partner abuse during pregnancy: a risk factor for low birth weight in Nicaragua. *Obstetrics and gynecology* 2002;100(4):700-5. [published Online First: 2002/10/18]
4. Devries K, Watts C, Yoshihama M, et al. Violence against women is strongly associated with suicide attempts: evidence from the WHO multi-country study on women's health and domestic violence against women. *Social science & medicine (1982)* 2011;73(1):79-86. doi: 10.1016/j.socscimed.2011.05.006 [published Online First: 2011/06/17]
5. Pallitto CC, Garcia-Moreno C, Jansen HA, et al. Intimate partner violence, abortion, and unintended pregnancy: results from the WHO Multi-country Study on Women's Health and Domestic Violence. *International journal of gynaecology and obstetrics: the official organ of the International Federation of*

For peer review only

Gynaecology and Obstetrics 2013;120(1):3-9. doi: 10.1016/j.ijgo.2012.07.003 [published Online First: 2012/09/11]
6. Brownell P. Neglect, abuse and violence against older women: Definitions and research frameworks (Review article). *South Eastern European Journal of Public Health* 2014;1
7. Mikton CR, Tanaka M, Tomlinson M, et al. Global research priorities for interpersonal violence prevention: a modified Delphi study. *Bulletin of the World Health Organization* 2017;95(1):36-48. doi: 10.2471/blt.16.172965 [published Online First: 2017/01/06]
8. Beaulaurier RLS, L. R.; Newman, F. L.; Dunlop, B. External barriers to help seeking for older women who experience intimate partner violence. *Journal of Family Violence* 2007;22(8):747-55.
9. Souto RQ, Guruge S, Merighi MA, et al. Intimate Partner Violence Among Older Portuguese Immigrant Women in Canada. *Journal of interpersonal violence* 2016 doi: 10.1177/0886260516646101 [published Online First: 2016/04/27]
10. Tetterton S, Farnsworth E. Older women and intimate partner violence: effective interventions. *Journal of interpersonal violence* 2011;26(14):2929-42. doi: 10.1177/0886260510390962 [published Online First: 2010/12/16]
11. Stockl H, Watts C, Penhale B. Intimate partner violence against older women in Germany: prevalence and associated factors. *Journal of interpersonal violence* 2012;27(13):2545-64. doi: 10.1177/0886260512436390 [published Online First: 2012/02/14]
12. Crockett C, Brandl B, Dabby FC. Survivors in the Margins: The Invisibility of Violence Against Older Women. *Journal of elder abuse & neglect* 2015;27(4-5):291-302. doi: 10.1080/08946566.2015.1090361 [published Online First: 2015/09/16]
13. McGarry J, Bowden D. Unlocking stories: Older women's experiences of intimate partner violence told through creative expression. *Journal of psychiatric and mental health nursing* 2017;24(8):629-37. doi: 10.1111/jpm.12411 [published Online First: 2017/07/07]
14. United Nations. Madrid International Plan of Action on Ageing. New York: United Nations 2002.
15. Department of Economic and Social Affairs. Neglect, Abuse and Violence against Older Women. New York: United Nations 2013.
16. Brownell P. A reflection on gender issues in elder abuse research: Brazil and Portugal. *Ciencia & saude coletiva* 2016;21(11):3323-30. doi: 10.1590/1413-812320152111.23142016 [published Online First: 2016/11/10]
17. Roberto KA, McPherson MC, Brossoie N. Intimate partner violence in late life: a review of the empirical literature. *Violence against women* 2013;19(12):1538-58. doi: 10.1177/1077801213517564 [published Online First: 2014/01/31]
18. Warmling D, Lindner SR, Coelho EBS. Intimate partner violence prevalence in the elderly and associated factors: systematic review. *Ciencia & saude coletiva* 2017;22(9):3111-25. doi: 10.1590/1413-81232017229.12312017 [published Online First: 2017/09/28]

For peer review only

19. Penhale B. Bruises on the soul: older women, domestic violence and elder abuse. *Bold : quarterly journal of the International Institute on Aging (United Nations - Malta)* 1998;8(2):16-30. [published Online First: 2002/09/26]
20. Crockett C, McCleary-Sills J, Cooper B, et al. Violence against Older Women: Violence against Women & Girls Resource Guide. . In: Bank TW, ed. Washington D.C.: The World Bank 2016.
21. McGarry JS, Christine. Domestic abuse and older women: exploring the opportunities for service development and care delivery. *The Journal of Adult Protection* 2011;13(6):294-301. doi: http://dx.doi.org/10.1108/14668201111194203
22. Yon Y, Mikton C, Gassoumis ZD, et al. The Prevalence of Self-Reported Elder Abuse Among Older Women in Community Settings: A Systematic Review and Meta-Analysis. *Trauma, violence & abuse* 2017:1524838017697308. doi: 10.1177/1524838017697308 [published Online First: 2018/01/16]
23. Bows H. Sexual Violence Against Older People: A Review of the Empirical Literature. *Trauma, violence & abuse* 2017:1524838016683455. doi: 10.1177/1524838016683455 [published Online First: 2018/01/16]
24. Yon Y, Mikton CR, Gassoumis ZD, et al. Elder abuse prevalence in community settings: a systematic review and meta-analysis. *The Lancet Global health* 2017;5(2):e147-e56. doi: 10.1016/s2214-109x(17)30006-2 [published Online First: 2017/01/21]
25. Yon Y, Ramiro-Gonzalez M, Mikton CR, et al. The prevalence of elder abuse in institutional settings: a systematic review and meta-analysis. *European journal of public health* 2018 doi: 10.1093/eurpub/cky093 [published Online First: 2018/06/08]
26. Yon Y, Mikton C, Gassoumis ZD, et al. Research Protocol for Systematic Review and Meta-Analysis of Elder Abuse Prevalence Studies. *Canadian journal on aging = La revue canadienne du vieillissement* 2017;36(2):256-65. doi: 10.1017/s0714980817000137 [published Online First: 2017/04/13]
27. Finfgeld-Connett D. Intimate partner abuse among older women: qualitative systematic review. *Clinical nursing research* 2014;23(6):664-83. doi: 10.1177/1054773813500301 [published Online First: 2013/09/21]
28. McGarry JA, Parveen; Hinchliff, Sharron. Older women, intimate partner violence and mental health: a consideration of the particular issues for health and healthcare practice. *Journal of Clinical Nursing* 2017;26(15/16):2177-91. doi: 10.1111/jocn.13490
29. Bohren MA, Hunter EC, Munthe-Kaas HM, et al. Facilitators and barriers to facility-based delivery in low- and middle-income countries: a qualitative evidence synthesis. *Reproductive health* 2014;11(1):71. doi: 10.1186/1742-4755-11-71 [published Online First: 2014/09/23]
30. Glenton C, Colvin CJ, Carlsen B, et al. Barriers and facilitators to the implementation of lay health worker programmes to improve access to maternal and child health: qualitative evidence synthesis. *The Cochrane database of systematic reviews* 2013(10):Cd010414. doi: 10.1002/14651858.CD010414.pub2 [published Online First: 2013/10/09]

For peer review only - http://bmjopen.bmj.com/site/about/guidelines.xhtml

31. Bohren MA, Vogel JP, Hunter EC, et al. The Mistreatment of Women during Childbirth in Health Facilities Globally: A Mixed-Methods Systematic Review. *PLoS medicine* 2015;12(6):e1001847; discussion e47. doi: 10.1371/journal.pmed.1001847 [published Online First: 2015/07/01]
32. Thomas J, Harden A. Methods for the thematic synthesis of qualitative research in systematic reviews. *BMC medical research methodology* 2008;8:45. doi: 10.1186/1471-2288-8-45 [published Online First: 2008/07/12]
33. Tong A, Flemming K, McInnes E, et al. Enhancing transparency in reporting the synthesis of qualitative research: ENTREQ. *BMC medical research methodology* 2012;12:181. doi: 10.1186/1471-2288-12-181 [published Online First: 2012/11/29]
34. Ministry of Health B. 2012 National Health Survey (NHS). . Thimphu, Bhutan.: Ministry of Health, 2012.
35. National Commission for the Advancement of Women - Lao PDR. Lao National Survey on Women's Health and Life Experiences 2014: A Study on Violence against Women. Lao PDR: National Commission for the Advancement of Women and Lao Statistics Bureau, 2015.

For peer review only

PubMed search

1. **Concept #1: Age – 50 and above**

Aged [MeSH Terms] OR Frail Elderly [MeSH Terms] OR senior* [TIAB] OR elderly [TIAB] OR Older [TIAB] OR later life [TIAB] OR above 50 [TIAB] OR above 60 [TIAB]

2. **Concept #2: Women**

Women [MeSH Terms] OR female [MeSH Terms] OR wife [TIAB] OR spouses [MeSH] OR wives [TIAB] OR “female partners” [TIAB] OR spouse [TIAB] OR spouses [TIAB]

3. **Concept #3: Violence**

Elder abuse [MeSH Terms] OR domestic violence [MeSH Terms] OR Intimate Partner Violence [MeSH Terms] OR battered women [MeSH] violence [MeSH Terms] OR aggression [MeSH Terms] OR spouse abuse [MeSH Terms] OR Physical Abuse [MeSH Terms] OR Rape [MeSH] OR elder neglect [TIAB] OR elder mistreatment [TIAB] OR elder maltreatment [TIAB] OR assault [TIAB] OR sexual abuse [TIAB] OR sexual assault [TIAB] psychological abuse [TIAB] or psychological violence [TIAB] OR emotional abuse [TIAB] or emotional violence [TIAB] or neglect [TIAB] or economic abuse [TIAB] or verbal abuse [TIAB] or violence against women [TIAB]

4. **Concept #4: Methodology**

(qualitative research methodology filter adapted from http://libguides.sph.uth.tmc.edu/search_filters/pubmed_filters and http://guides.lib.uw.edu/hsl/qualres/pubmed)

Qualitative research [MeSH Terms] OR Interviews as Topic [MeSH Terms] OR Focus Groups [MeSH Terms] OR Grounded Theory [MeSH Terms] OR Nursing Methodology Research [MeSH Terms] OR Anecdotes as Topic [MeSH Terms] OR Narration [MeSH Terms] OR Video Recording [MeSH Terms] OR Tape Recording [MeSH Terms] OR Personal Narratives as Topic [MeSH Terms] OR “semi-structured” [TIAB] OR semistructured [TIAB] OR unstructured [TIAB] OR structured [TIAB] OR informal [TIAB] OR “in-depth” [TIAB] OR indepth [TIAB] OR “face-to-face” [TIAB] OR “focus group” [TIAB] OR “focus groups” [TIAB] OR qualitative [TIAB] OR ethnograph* [TIAB] OR “key informant”[TIAB] OR thematic [TIAB] OR phenomenol* [TIAB] OR "grounded theory" [TIAB] OR "grounded study" [TIAB] OR "grounded studies" [TIAB] OR "grounded research" [TIAB] OR "grounded analysis"[TIAB] OR "grounded analyses"[TIAB] OR "life story" [TIAB] OR "life stories" [TIAB] OR "participant observation"[TIAB] OR "action research"[TIAB] OR "purposive sampling"[TIAB] OR "content analysis" [TIAB] OR discourse [TIAB] OR "narrative analysis" [TIAB]

5. **Combine: #1 AND #2 AND #4**

6. **Combine: #2 AND #3 AND #4**

For peer review only

BMJ Open

Violence against older women: A protocol for a systematic review of qualitative literature

Journal:	*BMJ Open*
Manuscript ID	bmjopen-2018-028809.R1
Article Type:	Protocol
Date Submitted by the Author:	18-Mar-2019
Complete List of Authors:	Meyer, Sarah; World Health Organization, Department of Reproductive Health and Research<br>Lasater, Molly; Johns Hopkins University Bloomberg School of Public Health, Mental Health<br>Garcia-Moreno, Claudia; World Health Organisation, Department of Reproductive Health and Research
**Primary Subject Heading**:	Qualitative research
Secondary Subject Heading:	Global health, Mental health, Public health
Keywords:	violence, older women, systematic review

SCHOLARONE™
Manuscripts

Violence against older women: A protocol for a systematic review of qualitative literature

Sarah R. Meyer[1], Molly E. Lasater[2], Claudia García-Moreno[1]

1. Department of Reproductive Health and Research, World Health Organization, Geneva, Switzerland.
2. Department of Mental Health, Johns Hopkins School of Public Health, Baltimore, USA.

Corresponding author: Dr Sarah Meyer, Department of Reproductive Health and Research, World Health Organization, smeyer@who.int, Phone number: 0041 22 791 2111, Fax number: 0041 22 791 4171

Key words: violence against women, systematic review, qualitative research,

Word count: 3,756

For peer review only

Abstract:

Introduction: There is sparse evidence globally concerning patterns of and types of violence against women aged 50 and older. Improved understanding of older women's experiences of violence, including types of violence, perpetrators, and health impacts, is needed to address evident gaps in the literature, address requirements for monitoring and reporting on global Sustainable Development Goal indicators, and inform policy and programming for preventing and responding to violence against older women. The aim of the systematic review is to identify, evaluate and synthesize qualitative studies from all countries, exploring violence against women aged 50 and above, identifying types and patterns of violence, perpetrators of violence, and impacts of violence on various health outcomes for older women.

Methods and analysis:

A systematic search for qualitative studies of violence against older women will be conducted in the following databases: PubMed, PsycINFO, Embase, CINAHL, PILOTS, ERIC, Social Work Abstracts, International Bibliography of the Social Sciences, Social Services Abstracts, ProQuest Criminal Justice, and Dissertations & Theses Global. Studies will be focused on violence against older women (aged 50 and above), using qualitative methodology, exploring women's experiences of any type of violence perpetrated by any type of perpetrator. Two authors will independently review titles and abstracts retrieved through the search strategy. Data extraction will be conducted independently by one author and quality assessment will be conducted by two authors, using an adapted version of the Critical Appraisal Skills Programme [CASP] scale. Data will be analysed and synthesised using a thematic synthesis approach.

Ethics and dissemination:

Ethics approvals are not required as primary data is not being collected. Findings will be disseminated through a publication in a peer-reviewed journal and used to inform development of a module to measure violence against older women, for use in specialized violence against women surveys.

Strengths and limitations of this study:

- This systematic review is designed with a comprehensive search strategy, to allow inclusion of all relevant qualitative studies of violence against older women globally;
- This review focuses on qualitative literature, in order to build understanding of lived experiences of older women subjected to different forms of violence.

For peer review only - http://bmjopen.bmj.com/site/about/guidelines.xhtml

- This systematic review uses established methods of systematic reviews of qualitative literature, including database searches, title and abstract screening, data extraction and data analysis;
- This review will use an adapted version of the Critical Appraisal Skills Programme [CASP] scale to assess quality of studies; and
- This review will utilize thematic analysis to synthesize findings from included studies and identify over-arching themes and sub-themes.

For peer review only

Introduction:

Violence against women [VAW] is a major public health problem, a gender inequality issue and a human rights violation. Violence has significant and long-lasting impacts on women's physical and mental health, including injuries, unintended pregnancy, adverse birth outcomes, abortions (often in unsafe conditions), HIV and sexually transmitted infections, depression, alcohol-use disorders and other mental health problems[1-5].

Much of this research has focused on women of reproductive age (15-49 years) as they suffer the brunt of partner violence and sexual violence. However, there is sparse evidence globally concerning patterns of and types of violence against women aged 50 and older[6], and this gap needs to be filled. A global research priority setting exercise on interpersonal violence indicated widespread consensus regarding the limited understanding of violence against older women. The exercise identified description of the nature, magnitude, distribution and consequences of elder abuse as priority research areas[7]. Specific risk and protective factors for violence victimization amongst women of reproductive age may not be relevant or applicable in the case of older women. For older women, different relationship dynamics may influence forms of abuse[8 9], recent exposure to violence may be interlinked with violence victimization at different stages of the lifecourse[10 11], and dynamics of ageing may influence decisions to disclose or report abuse[12]. Current lack of data on violence against older women may negatively impact service development and provision, including gaps in services, limited understanding of barriers to reporting and help-seeking amongst older women who are subjected to violence. It may also lead to inappropriate policy or programmatic responses to violence[10 13]. International policy frameworks, including the Madrid International Plan of Action on Ageing specifically identifies elimination of violence, neglect and abuse of older women as a priority, recognizing that older women's vulnerabilities are compounded by societal discrimination, poverty and lack of access to legal protections[14].

There are currently two dominant theoretical frameworks to understanding violence against older women, each linked to different definitions of violence and assumptions regarding measurement, prevention of violence and other interventions: older adult mistreatment and older adult protection[15 16 6]. The older adult mistreatment framework is informed by social gereontology, and understands violence as forms of elder abuse – focusing on age as the primary vulnerability to exposure to violence. The older adult protection framework specifically understands violence within the context of care-giving and institutional arrangements, where older adults' vulnerability to violence is a result

For peer review only - http://bmjopen.bmj.com/site/about/guidelines.xhtml

of reliance on caregivers. There is also an approach that focuses specifically on one form of violence affecting older women, namely intimate partner violence [IPV]. This approach adopts the definition of IPV used for women and girls below 50, and seeks to expand understanding of sexual, physical and psychological violence perpetrated by partners that older women may experience.

The lack of an over-arching framework for understanding violence against older women has resulted in literature and evidence that is fragmented, with some research focusing only on specific types of violence against older women (e.g., IPV)[17], some research (in particular, using the older adult mistreatment framework) lacking a focus on the gendered dimensions of violence[16 18], and some approaches that focus specifically on older women in protective settings and relationships with caregivers rather than also including women in community settings. A 2013 United Nations report on neglect, abuse and violence against older women noted that these divergent theoretical frameworks have stymied data collection and evidence generation efforts; for example, some studies (in particular, population-based studies of IPV) have focused on women of reproductive age, while other studies (for example, studies of women in care-settings) have excluded measurement of some types of violence, for example, perpetrated by intimate partners[15]. The elder abuse perspective has traditionally lacked understanding of the gendered nature, i.e., differences between women and men, of age-related vulnerabilities, for example, that women are less likely to have adequate pensions than men and that older women are more likely than older men to be financially dependent on family members[6 19 20]. Some researchers note that the term “elder abuse” may homogenise “older people rather than recognising individual differences, including gender”[21]. In the study of IPV, research has primarily focused on women of reproductive age, potentially leading to marginalization of older women’s experiences of IPV, which may differ in type and nature[12]. Researchers note that this conceptual split has resulted in “lack of attention of ageing issues in research on violence against women and, conversely, the lack of gender-based analysis in elder abuse research”[22]. Further, there is concern that these approaches primarily respond to the situation of older women in high-income, industrialized settings, and that forms of violence and vulnerability to violence more prevalent in low-income contexts are not adequately reflected in current literature, theoretical approaches and measurement methods[15 22]. Research is spread between disciplines and fields of inquiry, yet a more comprehensive understanding of violence against older women can be built by bridging these different approaches,[23] incorporating a perspective that is both age and gender-responsive[12]. Improved understanding of older women’s experiences of violence, including types of violence,

For peer review only - http://bmjopen.bmj.com/site/about/guidelines.xhtml

perpetrators, and health impacts, is needed to address evident gaps in the literature, address requirements for monitoring and reporting on global SDG indicators, and inform policy and programming for preventing and responding to violence against older women.

This manuscript describes a protocol for a systematic review that complements a previous systematic review of quantitative studies of elder abuse (against men and women aged 60+). That review found the global prevalence of elder abuse in community settings of men and women is 15.7% in the past year, with psychological abuse and financial abuse the most prevalent forms of abuse reported. The studies were very heterogenous and methodological variables were associated with prevalence, with larger sample size and random sampling associated with lower prevalence rates[24]. Sex was not found to be significantly associated with prevalence rates in the review, however, some studies did not provide sex-specific analysis. The prevalence of elder abuse in institutional settings may be as high as 64.2%, with data obtained from self-reports of perpetration by care-givers in institutions, with prevalence estimates highest for psychological abuse, and physical violence, neglect, financial abuse and sexual abuse less prevalent[25]. Findings from institutional settings indicate that being female is a significant risk factor for vulnerability to abuse. Analysis from the same review which focused on the findings specific to abuse of older women found a global prevalence of 14.1%, and similar to the analysis of men and women, psychological abuse was the most prevalent form of violence, followed by neglect. Financial abuse was less prevalent amongst women than in the analysis including both men and women[22]. Overall, there has been more work identifying and synthesizing the quantitative literature on elder abuse and violence against women[22 26], which may miss out several forms of abuse older women are exposed to, particularly in low- and middle-income countries. This confirms a need to rigorously synthesize the qualitative literature.

There are some existing systematic reviews relevant to this one; those reviews primarily focus on specific forms of violence against older persons or women, and some focus on qualitative literature. Warmling et al. conducted a systematic review of IPV against "elderly men and women", focusing on prevalence studies (cross-sectional, population-based studies) in any country and exploring factors associated with the experience of violence by men and women [18]. The exact age range constituting "elderly men and women" was not defined in the review. Findings included that psychological violence and economic abuse were the most prevalent forms of partner violence against older men and women, and alcohol-use, depression, low income, functional impairment and

previous exposure to violence were associated with this violence amongst older men and women. An empirical review of IPV in later life used a qualitative coding scheme to identify theoretical frameworks, conceptual themes and methodological approaches in the existing literature, examining 27 quantitative, 22 qualitative and 7 mixed-methods studies[17]. The review identified controlling behaviours and power dynamics in relationships that continue between intimate partners into later life, and may become further entrenched by caregiving dynamics. Similarly to the previous reviews, findings included that forms of IPV in later life shifted from a higher prevalence of physical and sexual abuse during reproductive age, to a higher prevalence of forms of psychological abuse, with results indicating "a shift from physical to non-physical forms of violence dominated late-life scenarios."[22] A review of qualitative research on IPV amongst older women identified a number of themes relevant to the dynamics of IPV against older women [27]. For example, women described patterns of abuse that were continuous and consistent with previous experiences of abuse in families of origin and previous relationships. However, patterns and types of IPV against older women were also described as shifting from predominantly physical violence experienced previously, to neglect, psychological violence and economic abuse becoming more prevalent. A systematic review and meta-synthesis of qualitative studies of IPV and older women focused on how previous exposure to IPV influenced health-seeking behaviours, specifically in the area of mental health care[28]. Bows conducted an empirical review of sexual violence against older people, including qualitative and quantitative studies focused specifically on sexual violence, with studies including any population of older adults (age range not defined), identifying widespread variation in prevalence rates across studies, with higher levels of sexual violence identified in studies using a domestic violence framework. The role of socio-demographic factors, such as ethnicity, marital status and living arrangements, were not consistently associated with violence victimization, and studies identified a range of perpetrators, primarily intimate partners or adult children[23]. Amongst these existing systematic reviews of qualitative literature, none have focused specifically on older women, while being inclusive of any form of violence.

The present systematic review will build on previous systematic reviews and strengthen the evidence-base by i) focusing specifically on women, or if including studies with women and men, only including studies that provide sex-stratified analyses; ii) focusing on *any* form of violence against women, rather than adopting a specific theoretical framework on what types of violence or perpetrators should be included from the outset iii) focusing on women aged 50 and above as many surveys often specifically

For peer review only - http://bmjopen.bmj.com/site/about/guidelines.xhtml

focus on women up to 49 years of age; and iv) focusing specifically on qualitative studies, to explore the nature and dynamics of violence against older women from the perspective of women and in low- and middle-income countries.

Aim:

The aim of this systematic review is to identify, evaluate and synthesize qualitative studies from all countries, exploring violence against women aged 50 and above, identifying types and patterns of violence, perpetrators of violence, and impacts of violence on various health outcomes for older women. The protocol is registered in PROSPERO (registration number CRD42019119467).

Methods:

The study was initiated in June 2018 and will be completed in May 2019.

Search strategy

We identified the following domains as part of the research question: age (50 and above); women; violence; and qualitative approach. For each of these domains, we identified the relevant keywords and search terms, which vary by database. Search strategy has been appropriately modified for each database, including syntax and specific terms, topics and/ or headings.

We will not limit the search by year of publication, language of publication or type of publication at the stage of searching the databases. We have consulted with a librarian to provide input into the tailored search strategy that we have developed for each database, and the search strategy has been informed by other relevant systematic reviews and established approaches to identifying qualitative literature. The finalized search strategy for PubMed is included in Supplementary Appendix 1.

We will also search reference sections of relevant existing systematic reviews to identify articles that fit inclusion and exclusion criteria. We have also identified experts in the field, including researchers, practitioners and policy makers, and have contacted them to provide any relevant literature. All experts will be contacted at least twice to provide the research team with additional resources to consider for inclusion.

Data sources

We will conduct searches in the following electronic databases: PubMed, PsycINFO, Embase, CINAHL, PILOTS, ERIC, Social Work Abstracts, International Bibliography of the

For peer review only

Social Sciences, Social Services Abstracts, ProQuest Criminal Justice, and Dissertations & Theses Global.

Data collection and analysis:

Eligibility criteria for the studies:

The inclusion criteria for studies to be considered to be included in this review are:

Inclusion criteria:

i. Women aged 50 and older (studies including men or including women younger than 50 will be included if sex-specific and age-specific analyses respectively are included. This age range was selected because 15-49 year is generally considered reproductive age and most current data and evidence on intimate partner violence is in this age group;
ii. Qualitative methodology (mixed methods studies will be included if the qualitative data analysis is presented separately); and
iii. Studies focused on women's experiences of **any type of violence** perpetrated by **any type of perpetrator.**

There are no language or date limits. The abstracts of articles not in English will be reviewed by team members fluent in that language where feasible.

Exclusion criteria:

i. Whole sample is children or adolescents (under 50 years)
ii. Sample of only men
iii. Only quantitative methodology
iv. Only includes perspective on violence against women reported by care providers, health professionals, nursing home managers.

Data management:

We will use EndNote V.X7.8 as our bibliographic software management platform. We will remove duplicates using EndNote, prior to exporting titles and abstracts to an Excel spreadsheet for review. Data extraction and quality assessment results will be recorded in separate Excel spreadsheets. A flow diagram will be presented in any final publications, showing results of each stage of the review and adhering to the PRISMA statement.

Selection of studies:

For peer review only

Two authors (SM, ML) will independently review titles and abstracts retrieved through the search strategy, to determine which should be included for full text review. If an abstract or title is considered relevant by either of the authors, it will be included for full text review. Two authors will independently review all articles selected for full text review for eligibility, to reach consensus on inclusion in the review. Any discrepancies will be resolved with the input of the third author (CGM). Reasons for excluding articles will be recorded.

Data extraction

After full text review, the following data will be extracted from all included articles using a standardized data extraction form: country studied, study design, research questions, sample size, characteristics of sample, data collection and analysis methods, main findings (as reported by the study's own authors), and any reported study limitations. Categories specific to the topic will also be included as data extraction progresses, including types and nature of violence, perpetrators of violence, and reported impacts of violence.

Data extraction will be conducted independently by one author, and accuracy of the data extraction checked by a second author, with discrepancies resolved by discussion to reach consensus. Reviewers will develop and pre-test a data extraction spreadsheet, to be used to compile a summary of characteristics and key findings of the included studies. The spreadsheet will also include categories relevant to data synthesis, described further below.

Quality appraisal

Risk of bias (quality) assessment:

Included studies will be assessed for quality using an adapted version of the Critical Appraisal Skills Programme [CASP] scale. The adapted scale, relevant for review of qualitative studies, will include the following domains: research aims, methodology, research design, recruitment strategy, data collection, data analysis, reflexivity, ethical considerations, findings, and research contributions [29]. The tool includes the following questions [30]:

1. Are the setting(s) and context described adequately?
2. Is the sampling strategy described, and is this appropriate?
3. Is the data collection strategy described and justified?
4. Is the data analysis described, and is this appropriate?
5. Are the claims made/findings supported by sufficient evidence?

For peer review only - http://bmjopen.bmj.com/site/about/guidelines.xhtml

6. Is there evidence of reflexivity?
7. Does the study demonstrate sensitivity to ethical concerns?
8. Any other concerns?

Two authors (SM, ML) will assess the quality of the studies and disagreement will be resolved either by discussion or inputs from the third reviewer (CGM). Studies will be assessed as having an overall quality of "high," "medium" or "low" based on evaluation according to the CASP categories. Quality assessment will not be used to determine if any studies should be excluded, but rather to assess the strength of each study and as part of the confidence assessment (see below).

Strategy for data synthesis

Findings will be presented and synthesized using a thematic synthesis approach. Following some descriptive analysis (for example, number of studies examining specific types of violence, which types of risks and outcomes are described in studies), two authors will utilize the findings extracted as part of the data extraction process to conduct initial open coding and develop broad themes to use to further sort the findings. The authors will use axial coding of the text units in order to develop first, second and third order themes [31 32]. The synthesis and all aspects of the systematic review process will be reported following the 21-item checklist provided in the Enhancing Transparency in Reporting the Synthesis of Qualitative Research statement[33]. If data allows, analyses comparing age groups (for example, 50-69 and 70+) and regions will be conducted.

Patient and public involvement:

Patients were not involved in the development of this systematic review. Public were not consulted specifically for the development of the research questions, however, previous research and consultations with experts has indicated that this is a fruitful and important area of research in the field of violence against women research.

Discussion:

This manuscript provides a description of a protocol for a systematic review of qualitative literature on violence against older women (aged 50 and above). Strengths of the proposed review include utilization of multiple databases and search strategies to ensure inclusion of as much of the relevant literature as possible; a clear and structured process of data extraction and quality assessment; and transparent method of data synthesis, adhering to guidelines on systematic reviews of qualitative literature[33]. This

systematic review of qualitative literature focuses on violence against older women, including any form of violence, and including studies conducted globally. It is an important complement to existing systematic reviews on the same and related topics, which have primarily included quantitative literature or focused only on IPV. Limitations include that studies that do not include perspectives of women affected by violence themselves are excluded. There is a significant literature documenting the perspectives of care-givers and health-care professionals on elder abuse and other forms of violence against women, however, to ensure a focus on the lived experiences of women subjected to violence, we will exclude studies that only include perspectives of caregivers and health care professionals.

There are two Sustainable Development Goal [SDG] Indicators directly related to violence against women: 5.2.1, which measures intimate partner violence [IPV] (Proportion of ever-partnered women and girls aged 15 years and older subjected to physical, sexual or psychological violence by a current or former intimate partner in the previous 12 months), and 5.2.2, which measures non-partner sexual violence [NPSV] (Proportion of women and girls aged 15 years and older subjected to sexual violence by persons other than an intimate partner in the previous 12 months). VAW prevalence studies, whether implemented as stand-alone or using a module as part of a Demographic and Health Survey, Reproductive Health or other type of survey, have primarily included women of reproductive age (15-49). Yet valid monitoring of and reporting on these SDG indicators requires data on older women to be collected and reported systematically. Some recent data collection efforts have sought to address this gap in data and evidence by including women aged 50 and above in studies using these standard approaches to measuring violence against women (for example, the national prevalence studies in Bhutan and Laos[34 35]). However, this approach to measurement of violence against older women – utilizing standard VAW survey measures, – may not adequately capture the specific violence experiences of older women. Women aged 50 and above may experience different types and patterns of violence, with risk factors and impacts on physical and mental health, that differ compared to women aged below 50. This systematic review will inform efforts to increase and improve availability and quality of data on violence against women aged 50 and above. This includes, for example, efforts led by the World Health Organization to develop or adapt a quantitative module to measure violence against older women. When we complete the systematic review, we will develop and refine a set of domains and themes that emerged from the data, to inform development of the module.

For peer review only - http://bmjopen.bmj.com/site/about/guidelines.xhtml

Ethics and dissemination:

Ethics approvals are not required as primary data is not being collected. We will present findings in a manuscript for publication in a peer-reviewed journal, through coordinated dissemination to researchers, practitioners, data users and generators with an interest in violence against women, and to experts selected to participate in an Expert Meeting on Violence against Older Women, to be convened by the Department of Reproductive Health and Research, World Health Organization.

Contribution: SM and CGM conceptualized and developed the systematic review. SM drafted the manuscript. ML and CGM read and provided substantive feedback on all sections of the manuscript.

Competing interest: There are no competing interests.

Funding: The systematic review is funded by the Department for International Development, UNWomen-World Health Organization Joint Programme on Strengthening Methodologies and Measurement and building national capacities for Violence against Women data. The funder had no role in developing the protocol.

For peer review only

References:

1. WHO. Global and regional estimates of violence against women: prevalence and health effects of intimate partner violence and non-partner sexual violence. Geneva: WHO 2013.
2. Ellsberg M, Jansen HA, Heise L, et al. Intimate partner violence and women's physical and mental health in the WHO multi-country study on women's health and domestic violence: an observational study. *Lancet (London, England)* 2008;371(9619):1165-72. doi: 10.1016/s0140-6736(08)60522-x [published Online First: 2008/04/09]
3. Valladares E, Ellsberg M, Pena R, et al. Physical partner abuse during pregnancy: a risk factor for low birth weight in Nicaragua. *Obstetrics and gynecology* 2002;100(4):700-5. [published Online First: 2002/10/18]
4. Devries K, Watts C, Yoshihama M, et al. Violence against women is strongly associated with suicide attempts: evidence from the WHO multi-country study on women's health and domestic violence against women. *Social science & medicine (1982)* 2011;73(1):79-86. doi: 10.1016/j.socscimed.2011.05.006 [published Online First: 2011/06/17]
5. Pallitto CC, Garcia-Moreno C, Jansen HA, et al. Intimate partner violence, abortion, and unintended pregnancy: results from the WHO Multi-country Study on Women's Health and Domestic Violence. *International journal of gynaecology and obstetrics: the official organ of the International Federation of Gynaecology and Obstetrics* 2013;120(1):3-9. doi: 10.1016/j.ijgo.2012.07.003 [published Online First: 2012/09/11]
6. Brownell P. Neglect, abuse and violence against older women: Definitions and research frameworks (Review article). *South Eastern European Journal of Public Health* 2014;1
7. Mikton CR, Tanaka M, Tomlinson M, et al. Global research priorities for interpersonal violence prevention: a modified Delphi study. *Bulletin of the World Health Organization* 2017;95(1):36-48. doi: 10.2471/blt.16.172965 [published Online First: 2017/01/06]
8. Beaulaurier RLS, L. R.; Newman, F. L.; Dunlop, B. External barriers to help seeking for older women who experience intimate partner violence. *Journal of Family Violence* 2007;22(8):747-55.
9. Souto RQ, Guruge S, Merighi MA, et al. Intimate Partner Violence Among Older Portuguese Immigrant Women in Canada. *Journal of interpersonal violence* 2016 doi: 10.1177/0886260516646101 [published Online First: 2016/04/27]
10. Tetterton S, Farnsworth E. Older women and intimate partner violence: effective interventions. *Journal of interpersonal violence* 2011;26(14):2929-42. doi: 10.1177/0886260510390962 [published Online First: 2010/12/16]
11. Stockl H, Watts C, Penhale B. Intimate partner violence against older women in Germany: prevalence and associated factors. *Journal of interpersonal violence* 2012;27(13):2545-64. doi: 10.1177/0886260512436390 [published Online First: 2012/02/14]
12. Crockett C, Brandl B, Dabby FC. Survivors in the Margins: The Invisibility of Violence Against Older Women. *Journal of elder abuse & neglect* 2015;27(4-

For peer review only - http://bmjopen.bmj.com/site/about/guidelines.xhtml

5):291-302. doi: 10.1080/08946566.2015.1090361 [published Online First: 2015/09/16]
13. McGarry J, Bowden D. Unlocking stories: Older women's experiences of intimate partner violence told through creative expression. *Journal of psychiatric and mental health nursing* 2017;24(8):629-37. doi: 10.1111/jpm.12411 [published Online First: 2017/07/07]
14. United Nations. Madrid International Plan of Action on Ageing. New York: United Nations 2002.
15. Department of Economic and Social Affairs. Neglect, Abuse and Violence against Older Women. New York: United Nations 2013.
16. Brownell P. A reflection on gender issues in elder abuse research: Brazil and Portugal. *Ciencia & saude coletiva* 2016;21(11):3323-30. doi: 10.1590/1413-812320152111.23142016 [published Online First: 2016/11/10]
17. Roberto KA, McPherson MC, Brossoie N. Intimate partner violence in late life: a review of the empirical literature. *Violence against women* 2013;19(12):1538-58. doi: 10.1177/1077801213517564 [published Online First: 2014/01/31]
18. Warmling D, Lindner SR, Coelho EBS. Intimate partner violence prevalence in the elderly and associated factors: systematic review. *Ciencia & saude coletiva* 2017;22(9):3111-25. doi: 10.1590/1413-81232017229.12312017 [published Online First: 2017/09/28]
19. Penhale B. Bruises on the soul: older women, domestic violence and elder abuse. *Bold : quarterly journal of the International Institute on Aging (United Nations - Malta)* 1998;8(2):16-30. [published Online First: 2002/09/26]
20. Crockett C, McCleary-Sills J, Cooper B, et al. Violence against Older Women: Violence against Women & Girls Resource Guide. . In: Bank TW, ed. Washington D.C.: The World Bank 2016.
21. McGarry JS, Christine. Domestic abuse and older women: exploring the opportunities for service development and care delivery. *The Journal of Adult Protection* 2011;13(6):294-301. doi: http://dx.doi.org/10.1108/14668201111194203
22. Yon Y, Mikton C, Gassoumis ZD, et al. The Prevalence of Self-Reported Elder Abuse Among Older Women in Community Settings: A Systematic Review and Meta-Analysis. *Trauma, violence & abuse* 2017:1524838017697308. doi: 10.1177/1524838017697308 [published Online First: 2018/01/16]
23. Bows H. Sexual Violence Against Older People: A Review of the Empirical Literature. *Trauma, violence & abuse* 2017:1524838016683455. doi: 10.1177/1524838016683455 [published Online First: 2018/01/16]
24. Yon Y, Mikton CR, Gassoumis ZD, et al. Elder abuse prevalence in community settings: a systematic review and meta-analysis. *The Lancet Global health* 2017;5(2):e147-e56. doi: 10.1016/s2214-109x(17)30006-2 [published Online First: 2017/01/21]
25. Yon Y, Ramiro-Gonzalez M, Mikton CR, et al. The prevalence of elder abuse in institutional settings: a systematic review and meta-analysis. *European journal of public health* 2018 doi: 10.1093/eurpub/cky093 [published Online First: 2018/06/08]

For peer review only

26. Yon Y, Mikton C, Gassoumis ZD, et al. Research Protocol for Systematic Review and Meta-Analysis of Elder Abuse Prevalence Studies. *Canadian journal on aging = La revue canadienne du vieillissement* 2017;36(2):256-65. doi: 10.1017/s0714980817000137 [published Online First: 2017/04/13]
27. Finfgeld-Connett D. Intimate partner abuse among older women: qualitative systematic review. *Clinical nursing research* 2014;23(6):664-83. doi: 10.1177/1054773813500301 [published Online First: 2013/09/21]
28. McGarry JA, Parveen; Hinchliff, Sharron. Older women, intimate partner violence and mental health: a consideration of the particular issues for health and healthcare practice. *Journal of Clinical Nursing* 2017;26(15/16):2177-91. doi: 10.1111/jocn.13490
29. Bohren MA, Hunter EC, Munthe-Kaas HM, et al. Facilitators and barriers to facility-based delivery in low- and middle-income countries: a qualitative evidence synthesis. *Reproductive health* 2014;11(1):71. doi: 10.1186/1742-4755-11-71 [published Online First: 2014/09/23]
30. Glenton C, Colvin CJ, Carlsen B, et al. Barriers and facilitators to the implementation of lay health worker programmes to improve access to maternal and child health: qualitative evidence synthesis. *The Cochrane database of systematic reviews* 2013(10):Cd010414. doi: 10.1002/14651858.CD010414.pub2 [published Online First: 2013/10/09]
31. Bohren MA, Vogel JP, Hunter EC, et al. The Mistreatment of Women during Childbirth in Health Facilities Globally: A Mixed-Methods Systematic Review. *PLoS medicine* 2015;12(6):e1001847; discussion e47. doi: 10.1371/journal.pmed.1001847 [published Online First: 2015/07/01]
32. Thomas J, Harden A. Methods for the thematic synthesis of qualitative research in systematic reviews. *BMC medical research methodology* 2008;8:45. doi: 10.1186/1471-2288-8-45 [published Online First: 2008/07/12]
33. Tong A, Flemming K, McInnes E, et al. Enhancing transparency in reporting the synthesis of qualitative research: ENTREQ. *BMC medical research methodology* 2012;12:181. doi: 10.1186/1471-2288-12-181 [published Online First: 2012/11/29]
34. Ministry of Health B. 2012 National Health Survey (NHS). . Thimphu, Bhutan.: Ministry of Health, 2012.
35. National Commission for the Advancement of Women - Lao PDR. Lao National Survey on Women's Health and Life Experiences 2014: A Study on Violence against Women. Lao PDR: National Commission for the Advancement of Women and Lao Statistics Bureau, 2015.

For peer review only

PubMed search

1. **Concept #1: Age – 50 and above**

Aged [MeSH Terms] OR Frail Elderly [MeSH Terms] OR senior* [TIAB] OR elderly [TIAB] OR Older [TIAB] OR later life [TIAB] OR above 50 [TIAB] OR above 60 [TIAB]

2. **Concept #2: Women**

Women [MeSH Terms] OR female [MeSH Terms] OR wife [TIAB] OR spouses [MeSH] OR wives [TIAB] OR “female partners” [TIAB] OR spouse [TIAB] OR spouses [TIAB]

3. **Concept #3: Violence**

Elder abuse [MeSH Terms] OR domestic violence [MeSH Terms] OR Intimate Partner Violence [MeSH Terms] OR battered women [MeSH] violence [MeSH Terms] OR aggression [MeSH Terms] OR spouse abuse [MeSH Terms] OR Physical Abuse [MeSH Terms] OR Rape [MeSH] OR elder neglect [TIAB] OR elder mistreatment [TIAB] OR elder maltreatment [TIAB] OR assault [TIAB] OR sexual abuse [TIAB] OR sexual assault [TIAB] psychological abuse [TIAB] or psychological violence [TIAB] OR emotional abuse [TIAB] or emotional violence [TIAB] or neglect [TIAB] or economic abuse [TIAB] or verbal abuse [TIAB] or violence against women [TIAB]

4. **Concept #4: Methodology**

(qualitative research methodology filter adapted from http://libguides.sph.uth.tmc.edu/search_filters/pubmed_filters and http://guides.lib.uw.edu/hsl/qualres/pubmed)

Qualitative research [MeSH Terms] OR Interviews as Topic [MeSH Terms] OR Focus Groups [MeSH Terms] OR Grounded Theory [MeSH Terms] OR Nursing Methodology Research [MeSH Terms] OR Anecdotes as Topic [MeSH Terms] OR Narration [MeSH Terms] OR Video Recording [MeSH Terms] OR Tape Recording [MeSH Terms] OR Personal Narratives as Topic [MeSH Terms] OR “semi-structured” [TIAB] OR semistructured [TIAB] OR unstructured [TIAB] OR structured [TIAB] OR informal [TIAB] OR “in-depth” [TIAB] OR indepth [TIAB] OR “face-to-face” [TIAB] OR “focus group” [TIAB] OR “focus groups” [TIAB] OR qualitative [TIAB] OR ethnograph* [TIAB] OR “key informant”[TIAB] OR thematic [TIAB] OR phenomenol* [TIAB] OR "grounded theory" [TIAB] OR "grounded study" [TIAB] OR "grounded studies" [TIAB] OR "grounded research" [TIAB] OR "grounded analysis"[TIAB] OR "grounded analyses"[TIAB] OR "life story" [TIAB] OR "life stories" [TIAB] OR "participant observation"[TIAB] OR "action research"[TIAB] OR "purposive sampling"[TIAB] OR "content analysis" [TIAB] OR discourse [TIAB] OR "narrative analysis" [TIAB]

5. **Combine: #1 AND #2 AND #4**

6. **Combine: #2 AND #3 AND #4**

For peer review only - http://bmjopen.bmj.com/site/about/guidelines.xhtml

For peer review only

PRISMA-P checklist and page numbers in manuscript:

Section and Topic	Checklist item number	Checklist item	Page number in manuscript [N/A if not applicable for qualitative systematic review protocol]
Title			
Identification	1a	Identify the report as a protocol of a systematic review	1
Update	1b	If the protocol is for an update of a previous systematic review, identify as such	N/A
Registration	2	If registered, provide the name of the registry (such as PROSPERO) and registration number	8
Authors			
Contact	3a	Provide name, institutional affiliation, e-mail address of all protocol authors; provide physical mailing address of corresponding author	1
Contributions	3b	Describe contributions of protocol authors and identify the guarantor of the review	13
Amendments	4	If the protocol represents an amendment of a previously completed or published protocol, identify as such and list changes; otherwise, state plan for documenting important protocol amendments	N/A
Support			
Sources	5a	Indicate sources of financial or other support for the review	13
Sponsor	5b	Provide name for the review funder and/or sponsor	13
Role of sponsor or funder	5c	Describe roles of funder(s), sponsor(s), and/or institution(s), if any, in developing the protocol	13

For peer review only

Introduction			
Rationale	6	Describe the rationale for the review in the context of what is already known	2-8
Objectives	7	Provide an explicit statement of the question(s) the review will address with reference to participants, interventions, comparators, and outcomes (PICO)	8
Methods			
Eligibility criteria	8	Specify the study characteristics (such as PICO, study design, setting, time frame) and report characteristics (such as years considered, language, publication status) to be used as criteria for eligibility for the review	9
Information sources	9	Describe all intended information sources (such as electronic databases, contact with study authors, trial registers or other grey literature sources) with planned dates of coverage	8-9
Search strategy	10	Present draft of search strategy to be used for at least one electronic database, including planned limits, such that it could be repeated	Supplementary mater
Study records			
Data management	11a	Describe the mechanism(s) that will be used to manage records and data throughout the review	9
Selection process	11b	State the process that will be used for selecting studies (such as two independent reviewers) through each phase of the review (that is, screening, eligibility and inclusion in meta-analysis)	10
Data collection process	11c	Describe planned method of extracting data from reports (such as piloting forms, done independently, in duplicate), any processes for obtaining and confirming data from investigators	10
Data items	12	List and define all variables for which data will be sought (such as PICO items, funding sources), any pre-planned data assumptions and simplifications	10
Outcomes and prioritization	13	List and define all outcomes for which data will be sought,	10

For peer review only

		including prioritization of main and additional outcomes, with rationale	
Risk of bias in individual studies	14	Describe anticipated methods for assessing risk of bias of individual studies, including whether this will be done at the outcome or study level, or both; state how this information will be used in data synthesis	10-11
Data synthesis	15a	Describe criteria under which study data will be quantitatively synthesised	N/A
Data synthesis	15b	If data are appropriate for quantitative synthesis, describe planned summary measures, methods of handling data and methods of combining data from studies, including any planned exploration of consistency (such as I^2, Kendall's τ)	N/A
Data synthesis	15c	Describe any proposed additional analyses (such as sensitivity or subgroup analyses, meta-regression)	N/A
Data synthesis	15d	If quantitative synthesis is not appropriate, describe the type of summary planned	11
Meta-bias(es)	16	Specify any planned assessment of meta-bias(es) (such as publication bias across studies, selective reporting within studies)	N/A
Confidence in cumulative evidence	17	Describe how the strength of the body of evidence will be assessed (such as GRADE)	11

For peer review only
